# New research on the global prevalence of female genital mutilation/cutting: Research, clinical, and policy implications

**DOI:** 10.1371/journal.pmed.1004096

**Published:** 2022-09-15

**Authors:** Kerrie Stevenson, Brenda Kelly

**Affiliations:** 1 Faculty of Public Health and Policy, London School of Hygiene and Tropical Medicine, London, United Kingdom; 2 Institute of Health Informatics, University College London, London, United Kingdom; 3 Oxford University Hospitals NHS Foundation Trust, The Oxford Rose Clinic, Oxford, United Kingdom

## Abstract

In this perspective, Kerrie Stevenson and Brenda Kelly discuss new research on the prevalence of female genital mutilation/cutting alongside clinical and policy implications.

Female genital mutilation or cutting (FGM/C) involves the partial or total removal of external female genitalia including other injuries to the female genital organs for nonmedical reasons [1]. The practice is considered a violation of a child’s human right to bodily integrity, a form of gender-based violence (GBV), and can result in severe health complications resulting in significant healthcare costs [2–4]. The United Nations Sustainable Development Goal (SDG) 5.3 calls for an end to the practice; however, one of the challenges in charting progress relates to establishing accurate baseline prevalence data within countries and regions [5].

In a recently published *PLOS Medicine* article, Farouki and colleagues present a global systematic review estimating the prevalence of FGM/C among women and girls [1,6,7]. The review includes 397,683 women across 28 countries, and 283,437 girls across 23 countries. Farouki and colleagues derive a global estimate of approximately 100 million women and girls affected, which is on the lower bound of previously published estimates, which range between 100 and 200 million [1,6,7]. This may be because some previous estimates included household-level prevalence studies that may not be nationally representative. The most common type of FGM/C among women was “flesh removed” (Type I or II) in 19 countries, and “not sewn closed” (Type I, II, or IV) among girls in 9 countries. The overall pooled prevalence of FGM/C among women aged 15 to 49 years was 38.3% (95% CI: 20.8% to 59.5%; PI: 0.48% to 98.8%) and 7.25% (95% CI: 3.1% to 16.0%; PI: 0.1% to 88.9%) among girls aged 0 to14 years. The prevalence among 0- to 14-year-old girls is likely underreported as they are still at risk at the point of surveying. Regardless, these data and others suggest the practice is less common in younger generations perhaps due to changing attitudes within communities in high-prevalence countries [8]. While FGM/C decreased for women and girls in 23 and 25 countries, respectively, some increases were reported in countries including Somalia, Burkina Faso, and Guinea-Bissau.

While this review advances our understanding of the global landscape of FGM/C, there are limitations to keep to in mind. One limitation stems from a lack of contextually valid survey tools for data relating to type of FGM/C. This may impact women and girls’ ability to identify the form of FGM/C performed. Additionally, women and girls may not be able to accurately recall which procedure was performed. The search strategy did not include labial elongation, which is another form of genital alteration often considered under Type IV FGM (defined as all other harmful procedures to the female genitalia for nonmedical purposes), but which does not involve flesh cutting, removal, or circumcision [9]. This is practised in eastern and southern Africa, including Uganda, Rwanda, Zambia, Zimbabwe, and Mozambique [9]. Although WHO removed labial elongation **from Type IV definition in the 2008 typology, the organisation and others recognised it could still be defined** a form of FGM, as it is a social convention that places pressure on young girls to change their genitalia and creates permanent genital changes [9]. The authors found a high prevalence of FGM/C among migrant women and interpret this as evidence that FGM/C appears to continue in those who migrate from countries where FGM/C is prevalent. However, caution should be exercised here as data from the United Kingdom and elsewhere suggest that most women with FGM/C underwent the procedure before arriving in their host country and that the practice is much less common in well-integrated migrant communities [10,11]. Their interpretation risks perpetuating negative and harmful stereotypes of migrant women, which may lend weight to racial profiling and disproportionate safeguarding measures [12].

This study collating the best available FGM/C prevalence evidence has important implications for researchers, clinicians, and policymakers. Farouki and colleagues highlight the need for expanded routine data collection in every country across the world, including globally agreed and locally validated surveillance tools [1,4]. Like other studies in this field, attempts to capture FGM/C prevalence are limited by the ability to obtain accurate data on a deeply embedded cultural practice that is illegal in many countries. Data were self-reported meaning prevalence could be underreported due to social desirability or legal implications. Furthermore, there are several countries for which no representative prevalence data exist but for which evidence from small-scale studies or anecdotal accounts indicate FGM/C is happening including Columbia, India, UAE, and Saudi Arabia [1]. Historic emphasis on harm reduction strategies has acted as an unintended driver of the medicalisation of FGM/C, which remains a major issue in countries such as Egypt, whereby parents and carers seek “safer” FGM/C rather than abandonment of the practice [13]. Medicalisation legitimises continuation of FGM/C practice and there is much that healthcare institutions and medical regulatory bodies could do to address this practice. We urge those working in this field to examine this study closely when considering future policy and research initiatives.

These data implore us to work with local communities to determine effective prevention strategies, particularly in countries with persistently high prevalence including Somalia and Egypt, and to learn from countries such as Ethiopia where prevalence appears to be falling. It is not possible to attribute the decline in Ethiopia to an individual approach, but evaluations of previous strategies stress the importance of multisectoral engagement rather than isolated programmes [14]. Strategies implemented include community conversations involving local faith leaders and policy and legal reform prohibiting FGM [14]. When planning future interventions, it is important to focus on the rights-based narrative of the child, including the importance of early education campaigns exploring gender equity and FGM [4,13]. Strategies coproduced with local communities are likely to be more effective than “anti-FGM/C” campaigns focussing on harm reduction [4,13]. FGM/C is a deeply embedded social norm, and success in reducing prevalence is often associated with grassroots community initiatives [4]. Effective examples include locally cocreated media and radio campaigns in Kenya and community engagement on the importance of formal education of women and girls in Ethiopia [14,15]. It should be noted that legal protection against FGM/C exists in most countries, and legislation may serve as deterrent in countries with a low prevalence of FGM/C owing to little societal support but will likely be ineffective in countries with a high prevalence as law enforcement officials may disregard legal consequences if the practice has societal value [1]. There is also a call to widen our gaze, recognising SDG 5 calls for an end to GBV and improving health equality for women and girls [5]. We must consider how the prevalence of FGM/C relates to other forms of GBV, low levels of female education, and child marriage to work to ensure emancipation for women and girls across the world.

## Abbreviations

FGM/C: female genital mutilation or cutting
GBV: gender-based violence
SDG: Sustainable Development Goal
